# Polylactide Composites with Mineral Fertilisers—Properties and Biodegradation

**DOI:** 10.3390/ma19030547

**Published:** 2026-01-29

**Authors:** Grzegorz Świderski, Marek Jałbrzykowski, Monika Kalinowska, Małgorzata Pawłowska, Grzegorz Markiewicz, Emilia Bujnowska, Agnieszka Z. Wilczewska, Jolanta Magnuszewska

**Affiliations:** 1 Department of Chemistry, Biology and Biotechnology, Bialystok University of Technology, Wiejska 45E, 15-351 Białystok, Poland; m.kalinowska@pb.edu.pl (M.K.); emilia.bujnowska@sspb.pl (E.B.); 2 Department of Materials Engineering and Production, Faculty of Mechanical Engineering, Białystok University of Technology, Wiejska 45A, 15-351 Białystok, Polandgrzegorz.markiewicz@sd.pb.edu.pl (G.M.); 3 Faculty of Environmenal Engineering and Energy, Lublin University of Technology, Nadbystrzycka 40B, 20-618 Lublin, Poland; 4 Department of Polymers and Organic Synthesis, Faculty of Chemistry, University of Białystok, Ciołkowskiego 1K, 15-245 Białystok, Poland; agawilcz@uwb.edu.pl (A.Z.W.);

**Keywords:** polylactide, PLA, composites, biodegradation, fertilisers

## Abstract

**Highlights:**

- Additives strongly affect the decomposition rate of polylactide-based composites.
- Biopolymer degradation is dependent on environmental conditions.
- Mineral fertiliser components alter the mechanical performance of PLA–starch composites.

**Abstract:**

Polylactide (PLA) composites were prepared and doped with starch (10% by weight), and mineral salts used as mineral fertilisers (MgSO_4_, KNO_3_, Ca(NO_3_)_2_ and Ca_3_(PO_4_)_2_) were prepared. The content of the added fertilisers was 2% by mass in the composites. The tensile strength properties of the obtained composites were tested. The effect of the addition of fertilisers on the structure of polylactide was analysed using spectroscopic methods (FTIR and FTRaman). The thermal properties of the obtained composites were tested using thermogravimetry (TG/DTG) and differential scanning calorimetry (DSC). PLA composites with fertilisers were tested for biodegradability in two types of soil—field soil and horticultural soil—and in compost. Biodegradability was assessed based on the mass loss of biodegraded composites, spectroscopic tests and visual assessment of changes occurring in the composites. Tests were performed on the respiratory activity of microorganisms in the compost extract in which the tested composites were placed. The addition of mineral salts used in the tested composites significantly influenced the biodegradation rate of the composites. Mineral compounds (MgSO_4_, KNO_3_ and Ca(NO_3_)_2_) added to the PLA–starch composite improve its mechanical properties. It should also be noted that the addition of mineral salts to the prepared composites did not affect the chemical structure of polylactide. The addition of mineral salts to PLA also did not significantly affect its thermal properties, as demonstrated by DSC and TG thermal analysis.

## 1. Introduction

Biodegradable plastics such as polylactide (PLA), polyglycolic acid, polycaprolactone and polyhydroxybutyrate have been used to produce implant composites, packaging, compostable bags, biodegradable cutlery and tableware, as well as for the controlled release of drugs, pesticides and fertilisers [1,2,3]. The biodegradation time of polylactide, like other polymers, depends on many factors, including molecular weight, crystallinity, purity and external factors (pH, environmental humidity, presence of microorganisms, aerobic and anaerobic conditions and presence of salts and inorganic compounds) in the environment in which the polymer decomposes [4,5,6,7].

**Figure 1:**
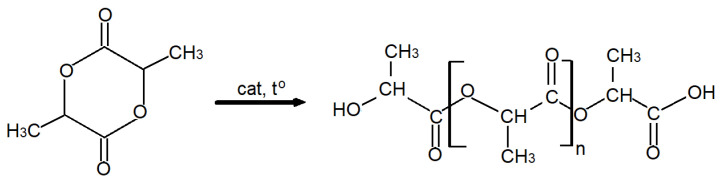
Synthesis of polylactide in the polymerisation process.

**Figure 2:**
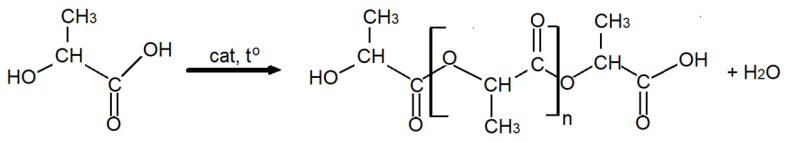
Synthesis of polylactide in the polycondensation process.

**Figure 3:**
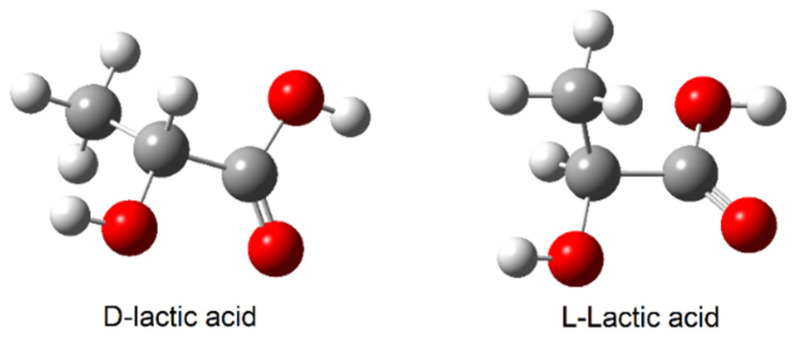
Structures of D-lactic acid and L-lactic acid.

**Figure 4:**
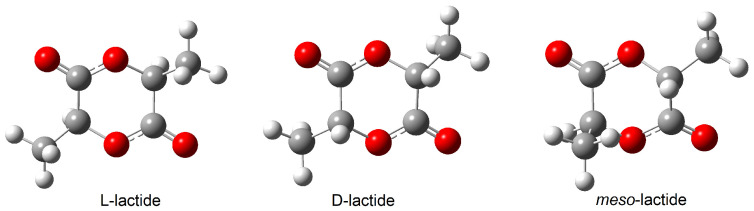
Structures of L-lactide, D-lactide and *meso*-lactide.

PLA is a biodegradable polymer produced by ring-opening polymerisation of lactide (Figure 1) or by polycondensation of lactic acid (Figure 2) [8,9,10]. Lactic acid occurs in the form of two isomers, L-lactic acid and D-lactic acid (Figure 3). Dilactide can form three isomers (Figure 4). The monomer, lactic acid, is produced on an industrial scale by fermenting plant materials such as corn and potatoes [11].

There are three stereoisomers of PLA: poly(L-lactide) (PLLA), poly(D-lactide) (PDLA) and poly(D,L-lactide) (PDLLA) [12]. PLA is competitive among biodegradable polymers for composite applications due to its low production costs, high mechanical strength and stiffness as well as water resistance. However, its melting point (175 °C) is close to its thermal degradation temperature (200 °C), which limits its processing [13].

Pure polylactide biodegrades slowly in the environment. PLA degradation occurs through hydrolysis and chain scission. The presence of alkyl groups hinders water attack and slows hydrolysis [14,15]. Furthermore, PLA has been shown to be relatively resistant to attack by microorganisms in soil as soil bacteria produce few natural enzymes that degrade this polymer [16]. Polylactide biodegraded rapidly in anaerobic or aerobic environments at temperatures above 60 °C (above the glass transition temperature) [16,17]. Through appropriate modifications of PLA polymer and the use of various additives, it is possible to obtain composites with different properties and different rates of biodegradation [18,19,20,21]. Various chemical, physical and biological processes may be involved in PLA degradation. Environmental factors not only affect the polymer to be degraded but also have a key impact on the microbial population and the activity of the microorganisms themselves that are involved in biodegradation [18]. From a biological perspective, the degradation of polylactide involves enzymatic mechanisms associated with microorganisms capable of producing extracellular hydrolytic enzymes. Enzymes such as esterases, lipases, proteases and others play a key role in PLA biodegradation by catalysing the hydrolysis of ester bonds in the polymer backbone [22,23,24,25]. These enzymes are secreted by various bacteria and fungi, including species of *Bacillus*, *Pseudomonas*, *Amycolatopsis* and *Aspergillus*, which have been reported to exhibit PLA-degrading activity under suitable environmental conditions [26,27,28,29]. The efficiency of enzymatic degradation depends strongly on polymer characteristics such as molecular weight, crystallinity, stereoregularity and surface hydrophilicity, as well as on environmental factors affecting enzyme activity, including temperature, moisture, pH and nutrient availability [30,31,32]. Enzymatic hydrolysis typically precedes microbial assimilation, leading to the formation of oligomers and lactic acid, which can subsequently be metabolised by microorganisms as carbon and energy sources [15,33,34,35]. Due to its relatively rapid decomposition rate, polylactide is used to produce biocomponents, the usefulness of which is related to the release of desired substances. One such application is the production of composites that contain slow-release fertilisers for the soil. Commercially, fertiliser granules in biodegradable polymer coatings, which slowly release minerals into the soil, have been used. Another application could be the production of biodegradable polymer pots using mineral fertilisers, which could be used in horticulture. Biodegradable pots for the commercial market are mainly produced from natural, decomposable materials such as peat, wood fibres, cellulose or biopolymer blends. After a certain period of time, these materials decompose in the soil, enriching it with organic components. Enriching such products with mineral fertilisers requires the use of polymer materials with higher durability, such as PLA. Research on the biodegradation of polymers doped with fertilisers, especially in terms of biodegradation conditions, the rate of decomposition in the soil and the release of mineral salts, is extremely important here. The aim of the study was to examine the properties of biocomposites consisting of PLA and starch mixed with selected inorganic salts commonly used as components of mineral fertilisers in agriculture. The study examined the mechanical and physicochemical properties of composites consisting of PLA, starch and fertilisers, as well as their rate of biodegradation in soils with different properties—varying pH, density and moisture content.

## 2. Materials and Methods

### 2.1. Prepared PLA Composites

The composites tested consisted of polylactide doped with starch (soluble starch, analytical grade, WE: 232-686-4) (Chempur, Piekary Śląskie, Poland) (10% by weight) and mineral fertiliser in an amount of 2% by weight. The composites were doped with the following mineral salts used as fertilisers: magnesium sulphate (MgSO_4_) (Chempur, Poland), calcium phosphate Ca_3_(PO_4_)_2_ (Chempur, Poland), calcium nitrate (Ca(NO_3_)_2_ (Chempur, Poland) and potassium nitrate (KNO_3_) (Chempur, Poland). Polylactide granules (Biopolymer 4043D) (NatureWorks, Blair, NE, USA) were used to prepare the composites. The weighed amounts of PLA granules, starch and fertiliser were mixed by hand and then extruded using a ZAMAK EHP 25 ELINE extruder (Zamak Mercator, Skawina, Poland). The temperatures in the individual zones of the device were 80 °C, 140 °C and 160 °C (from the feed zone to the head) with an extrusion speed of 10 rpm. The materials obtained in the pressing process were then crushed using a SHINI mill. After crushing, the material was granulated and fed into an injection-moulding machine. A BORCHE BS60 (Borche, Guangzhou, China) injection-moulding machine was used to produce fittings in accordance with PN-EN ISO 527 (Geneva, Switzerland, 2019). The injection temperatures were 190 °C, 200 °C, 190 °C and 180 °C (from the injection nozzle to the feed zone). The cooling time was 25 s.

**Figure 5:**
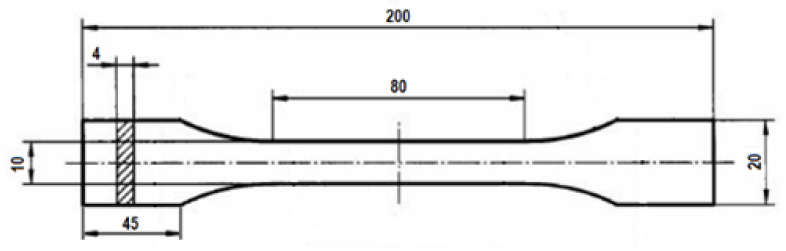
Dimensions of extruded PLA composite samples.

Pure polylactide biodegrades relatively slowly; it is a relatively hard and stiff material. The addition of a filler, such as starch, significantly accelerates the biodegradation process of polylactide but also weakens the mechanical properties of the polymer. A polymer with more than 20% starch becomes quite brittle and fragile, as indicated by the results from our preliminary research, so the composites containing 10% starch were used for the study. The parameters of the prepared samples are shown in Figure 5.

**Figure 6:**
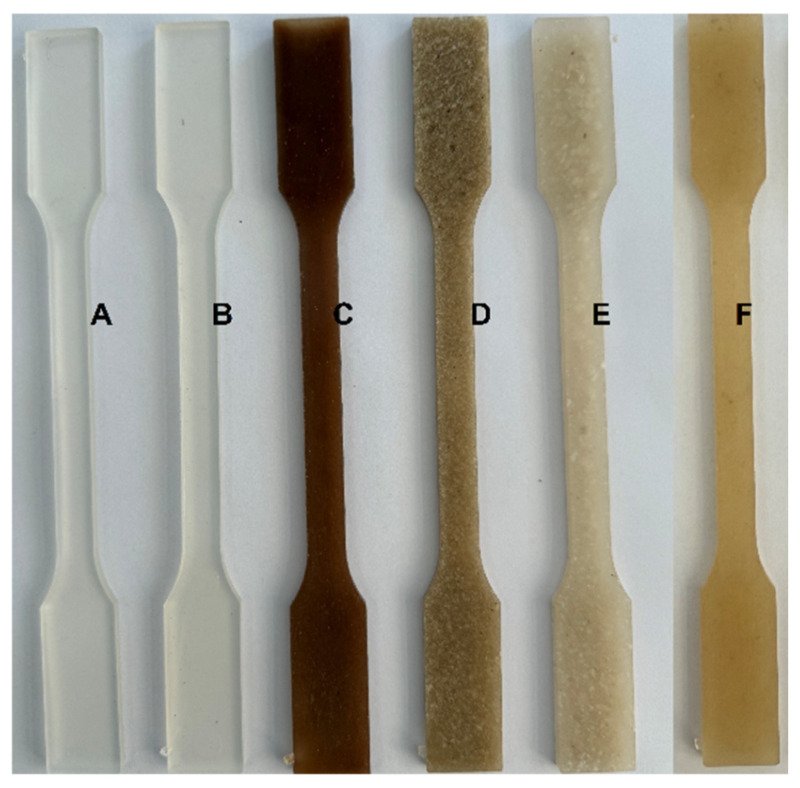
Photographs of PLA composite samples with additives: A—pure PLA; B—PLA + starch; C—PLA + starch + Ca(NO_3_)_2_; D—PLA + starch + Ca_3_(PO_4_)_2_; E—PLA + starch + MgSO_4_; F—PLA + starch + KNO_3_.

Figure 6 shows photographs of the PLA composite samples produced with starch and fertilisers.

**Figure 7:**
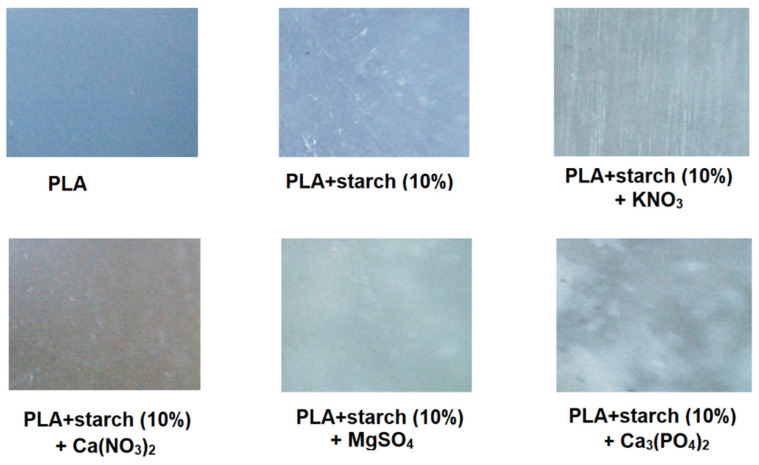
Microscope photo of surface PLA and their composites.

The 50-fold magnification photographs of the prepared composite samples were taken using a model NICOLET IS50-FT-RAMAN (Thermo Scientific, Madison, WI, USA) Raman microscope (Figure 7). The photographs show the uniform distribution of additives (starch and fertilisers) in the polylactide mass in the prepared composites.

### 2.2. Strength Tests

Tensile strength tests were performed in accordance with ISO 527 using a Zwick/Roell Z010 testing machine (ZwickRoell GmbH & Co. KG, Ulm, Germany). Three strength measurements were taken for each composite.

### 2.3. FTIR and Raman Spectroscopy

FTIR studies were conducted using a Bruker alpha FTIR (Bruker, Mannheim, Germany) spectrophotometer with an ATR multi-reflection measurement attachment. Measurements were performed in the range of 3400–600 cm^−1^ with a resolution of 1 cm^−1^. Raman spectra were recorded using a NICOLET IS50-FT-RAMAN device in the range of 3400–600 cm^−1^ with a resolution of 1 cm^−1^.

### 2.4. Thermal Decomposition (TG/DTG) and Differential Scanning Calorimetry (DSC)

#### 2.4.1. TG/DTG Study

Firstly, 3 mg of sample was weighed in the measuring ceramic cell. Then, the cell was consolidated using a hydraulic press. An empty cell was used as the reference cell. The reference cell was also consolidated using a hydraulic press. The measurements were performed on a METTLER TOLEDO STAR^®^ (Mettler-Toledo AG, Analytical, Schwerzenbach, Switzerland) instrument in the temperature range from 50 to 900 °C with a temperature increase of 10 K/min.

#### 2.4.2. DSC Study

The measurements were performed on a METTLER TOLEDO STAR^®^ instrument in the temperature range from 0 to 250 °C, temperature increase: 10 K/min, measuring cell: Al (aluminium).

### 2.5. Biodegradability in Soils

The biodegradation of PLA composites with fertilisers was tested in two types of soil (horticultural soil and field soil) and in compost. Commercially available universal garden soil and soil taken from the cultivated areas (around Białystok, Poland) were used in the study. The samples of field soil were collected from the upper horizon (10–30 cm) of the arable soil which had been fallowed for a year. The soil was cleared of roots. Compost produced from green waste came from the Waste Management Plant (Hryniewicze, near Białystok). The soils and compost were characterised beforehand. The pH of the soils and compost was measured using a pH meter (FiveEasy Metler Toled). The moisture content of the soils and compost was determined using the drying method by weighing and drying at 105 °C to a constant weight. The water content was determined from the difference in weight. Measurements were taken for three independent samples for each type of soil and compost. Soil density was determined using the pycnometer method and (using a La Chatelier flask). The carbon and nitrogen content was examined using a multi N/C analyser (Multi NC DUO (ChD) Analytik Jena, Jena, Germany). The determination of selected elements in soil compost samples was performed by X-ray spectrofluorimetry on a Bruker S2 PICOFOX apparatus (Bruker, Germany).

Biodegradation tests were conducted in 1 L polypropylene containers. The containers were filled with a fixed amount of soil/compost, and composite samples of similar dimensions as well as weights were placed in the soil. The experiment lasted 9 weeks. At weekly intervals, the samples were removed from the soil, cleaned and dried with a stream of cold air then weighed and photographed, and FTIR spectra were recorded. The samples were then returned to the soil. Three independent experiments were performed for each composite sample. The water lost from the soil due to evaporation during the experiment was replenished. The soil moisture in the samples was controlled by measuring the mass of water loss and supplemented accordingly to the initial water amount.

### 2.6. Preparation for Testing in Compost Extract

The biodegradability of the obtained composites was assessed using a standard method involving the measurement of oxygen consumption by microorganisms. The OxiTOP 12 (Xylem Analytics, Weilheim, Germany) measuring system was used for the tests. The tests were carried out in a suitably prepared compost extract. Composite samples measuring 10 mm × 60 mm × 40 mm were placed in the compost solution. Each measuring bottle was filled with 250 cm^3^ of compost extract, 4 drops of NT600 nitrification inhibitor were added and then a magnetic stirrer was immersed in it. Next, a rubber carrier with a carbon dioxide absorber (0.4 g NaOH) was placed in each bottle. Finally, OxiTop measuring heads were attached, and the closed bottles were placed on a mixing platform and put into a thermostatic cabinet. Incubation was carried out for 7 days at 20 °C. The Oxi TOP controllers were set to the BOD mode with measurements taken every 24 h for 7 days.

The compost extract used in the test was prepared according to the method of Richert et al. [36]: 900 mL of Ringer’s solution with the following composition was added to 100 g of mature compost: 2.250 g sodium chloride, 0.105 g potassium chloride, 0.120 g calcium chloride and 0.050 g sodium bicarbonate. The suspension prepared in this way was homogenised in a laboratory mechanical homogeniser (5000 rpm, *t* = 5 min) and then filtered through a sterile linen cloth. The resulting solution was used as a suspension of microorganisms for testing.

## 3. Results and Discussion

### 3.1. The Influence of Fertilisers Added to PLA on the Physical Properties of the Obtained Composites

Table 1 summarises the results of tensile strength measurements of PLA composites with starch and mineral fertilisers.

Pure polylactide exhibits a tensile strength of 67.77 MPa and an elongation at break of 1.77%. The addition of 10% starch significantly reduced the mechanical properties of the polymer. A composite composed of PLA and 10% starch demonstrated a strength of 35.33 MPa (a ~48% decrease compared to pure polylactide). Elongation at break decreased to 0.73%, while stress at break decreased to 13.80 MPa. The addition of starch significantly deteriorated the mechanical properties of PLA. Addition of some mineral fertilisers can partially reduce this negative influence. The best modifier is calcium nitrate, which significantly increases the strength and resistance of the composite (PLA + starch). The strength of the PLA + starch + Ca(NO_3_)_2_ composite increases to 48.23 MPa, which is significantly higher than for PLA + starch alone. The stress at break is also higher (18.57 MPa) than for the PLA + starch composite. The PLA + starch + MgSO_4_ composite has a tensile strength of 36.10 MPa—similar to the PLA + starch composite. The stress at break for this composite is slightly higher than that of the PLA + starch composite (14.10 MPa). The addition of KNO_3_ also improves the mechanical properties of the PLA + starch composite (strength: 40.10 MPa, stress at break: 15.67 MPa, elongation: 0.80%). Contrary to the previously mentioned mineral salts, the addition of Ca_3_(PO_4_)_2_ to PLA + starch composite decreased the tensile strength (29.13 MPa), elongation at tensile strength (0.63%), stress at failure (11.37 MPa) and elongation at failure (0.67%). The values of these parameters for this composite were the lowest compared to the other materials.

### 3.2. FTIR and Raman Spectroscopy of PLA Composites

**Figure 8:**
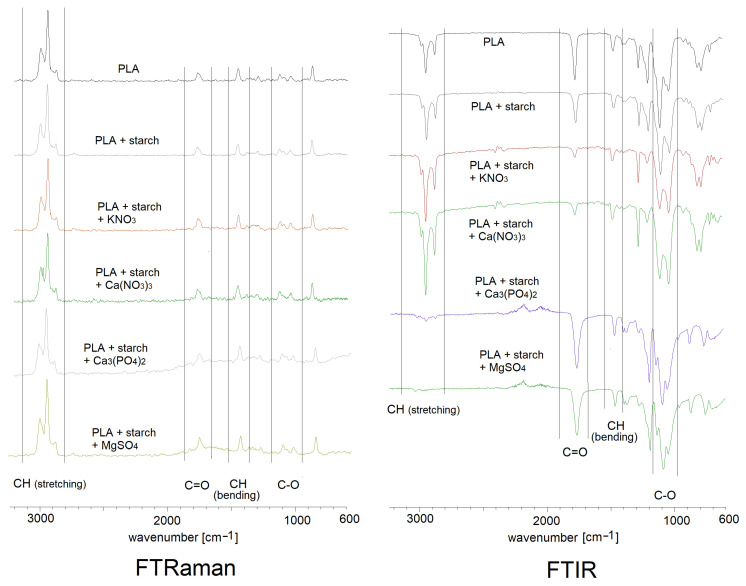
FTIR ATR and Raman spectra registered for PLA composites.

Figure 8 shows FTIR infrared spectra recorded using the ATR technique and spectra recorded using the Raman technique for pure polylactide and polylactide composites doped with starch and mineral fertilisers. Table 2 summarises the main bands and their assignments occurring in the recorded spectra of PLA and its composites. The spectra were assigned based on literature data [37].

The FTIR and Raman spectra of polylactide show a series of characteristic bands originating from C-H stretching vibrations in the methyl groups of the polymer. These include asymmetric stretching vibration bands ν_as_CH_3_ present in the FTIR spectrum at wavenumbers 3001 cm^−1^ and 2930 cm^−1^ and in the Raman spectrum at wavenumbers 3002 cm^−1^ and 2947 cm^−1^. The asymmetric and deformation C-H stretching vibration bands in the methyl group were observed in the FTIR spectrum at wavenumbers 1456 cm^−1^ and in the FT-Raman spectrum at a wavenumber of 1455 cm^−1^. The bands located at 1383 cm^−1^ in the FTIR spectrum and at 1390 cm^−1^ in the Raman spectrum correspond to the deformation vibrations of the CH_3_ methyl group. The bands associated with the stretching vibrations of the carbonyl group C=O were observed in the infrared spectrum at wavenumbers 1750 cm^−1^ and 1771 cm^−1^ in the Raman spectrum and in the wavenumber ranges 754 cm^−1^, 698 cm^−1^ (FTIR) and 761 cm^−1^, 691 cm^−1^ (Raman). The spectra also show vibration bands originating from the stretching of C-C bonds (in the CH-CH_3_ group) at wavenumbers of 1361 cm^−1^ (FTIR spectrum) and 1360 cm^−1^ (Raman spectrum). Stretching bands in the C-O-C ester group appear in the FTIR spectrum at 1266 cm^−1^ and at 1298 cm^−1^, 1257 cm^−1^ in the Raman spectrum. Deformations of the C-O-C ester group can be observed in the spectra in the form of bands located at 1181 cm^−1^ and 1078 cm^−1^ (FTIR) and 1184 cm^−1^ and 1098 cm^−1^ (Raman).

The positions of the bands in the FTIR and Raman spectra of the polymers doped with starch and mineral fertilisers do not differ significantly from the positions of the corresponding bands in the spectrum of pure PLA (Figure 8, Table 2). No chemical interactions between the metal ions contained in the added mineral salts and polylactide were observed (the C=O carbonyl group bands do not undergo significant shifts in the spectra).

### 3.3. Thermal Analysis TG/DTG and DSC

**Figure 9:**
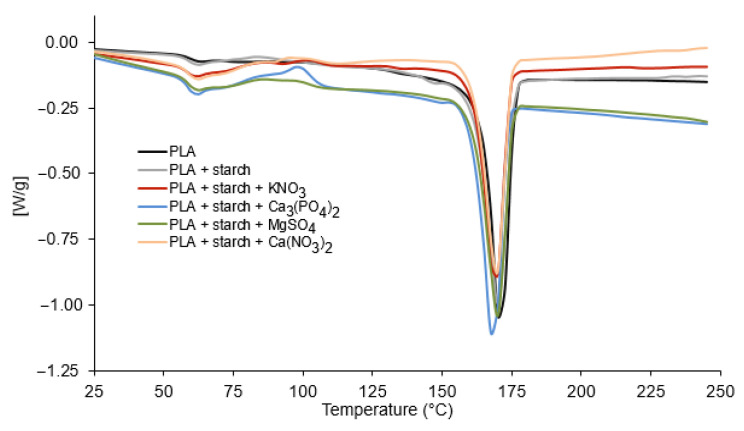
DSC curves for polylactide and its composites with starch and mineral fertilisers.

**Figure 10:**
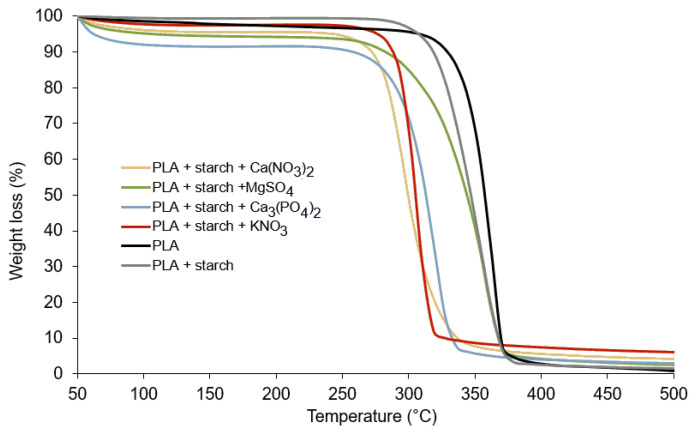
TG curves for polylactide and its composites with starch and mineral fertilisers.

**Figure 11:**
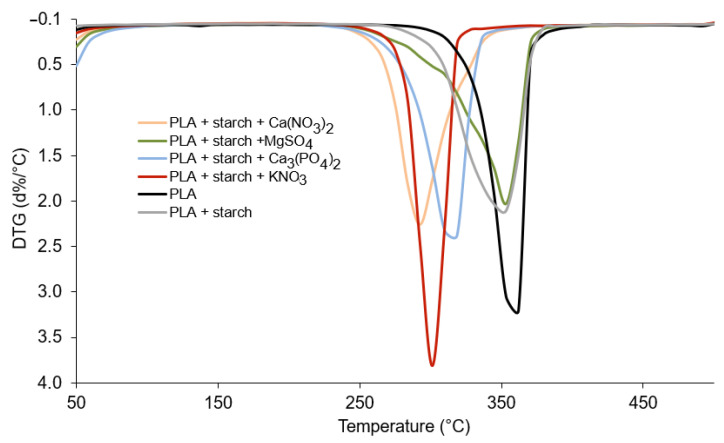
DTG curves for polylactide and its composites with starch and mineral fertilisers.

Thermal analysis TG/DTG and differential scanning calorimetry (DSC) show that polylactide undergoes a cycle of changes when heated from room temperature to high temperatures (500 °C) (Figure 9, Figure 10 and Figure 11). At 65 °C, the polymer undergoes a glass transition [38,39], which can be observed on the DSC curve in the form of an endothermic peak (Figure 9, Table 3). The addition of starch and fertilisers slightly lowers the glass transition temperature of PLA to 62.5 °C (Table 3) and increases the intensity of the process (Figure 9). At 82.5 °C, the polymer crystallises (exothermic peaks on the DSC curve). The addition of starch (10%) does not affect the temperature of the process, while the addition of mineral fertilisers causes a significant increase in the temperature of this process. For the Ca_3_(PO_4_)_2_-doped polymer, the temperature increased up to 97.5 °C and for the other composites to slightly lower values. The crystallisation temperature of polylactide is affected by the addition of mineral compounds and the presence of water, which comes from the hydrated salts used to dope the composite. No effect of the additives used on the melting temperature of polylactide, which is 170 °C (peaks on endothermic DSC curves, Figure 9), was observed, similarly to the melting temperature of the composites. Such minimal changes in the thermal transitions confirm that the chemical structure of PLA remains unchanged upon addition of mineral salts and starch and that the effects observed are related primarily to physical interactions and the presence of water in hydrated salts.

The thermal decomposition of PLA begins at 290 °C, and a sharp jump (mass loss) can be observed on the TG curve (Figure 9) up to a temperature of 380 °C (Table 4). The mass loss is 96.50%. Above 390 °C to 500 °C (the final temperature of the process), the residues from the thermal decomposition of PLA slowly burn out. At 500 °C, 99.20% of the initial mass is lost. The decomposition of PLA mixed with starch (10%) proceeds in a similar manner to the decomposition of pure PLA, with the decomposition process starting at a slightly lower temperature—275 °C. The addition of mineral fertilisers lowers the decomposition temperature of polylactide (Table 4). It has been observed that in the temperature range from approximately 50 °C to 200 °C, during the decomposition of PLA doped with mineral fertilisers, there is a loss of mass ranging from 2.70% for PLA with KNO_3_ to 8.45% (the greatest loss of mass in the case of PLA with the addition of Ca_3_(PO_4_)_2_). The weight loss is partly due to the loss of water contained in the mineral salts used as additives and the initial decomposition of the PLA polymer and starch. Above the decomposition temperature of the tested composites, the decomposition residues slowly burn. In the case of the composites doped with mineral salts, residues ranging from 2.50 to 5% were observed after the process was completed. The mineral salts used are characterised by relatively high decomposition temperatures, which is why they did not decompose within the temperature range used in the process. The TG results clearly indicate that the addition of mineral salts does not cause chemical degradation or destabilisation of the PLA polymer chain during thermal processing. The observed differences in decomposition temperatures and residue amounts are consistent with the physical presence of hydrated salts and mineral residues. These findings align with the FTIR and Raman spectroscopy data, which showed no changes in the chemical structure of PLA after doping with salts. Moreover, the thermal stability of PLA during processing at temperatures above 180 °C is confirmed, supporting the suitability of the chosen processing conditions for composite preparation without inducing polymer degradation. The results of thermal analysis (TG/DSC) indicate that the main degradation of PLA occurs within a temperature range very similar to that of neat PLA, with no observable shift of the characteristic chemical degradation stage and no additional decomposition steps. The minor differences observed in the onset decomposition temperatures and in the low-temperature mass loss region are primarily attributed to dehydration of the salts and their physical presence within the polymer matrix, rather than to catalytic degradation of the polymer chains.

The DTG curve of neat PLA shows a single sharp degradation peak at about 360 °C, indicating a one-step thermal decomposition. The addition of starch shifts the peak to lower temperatures and broadens it, reflecting reduced thermal stability and overlapping degradation of PLA and starch. Incorporation of mineral fertilisers further accelerates degradation, with nitrate salts (especially KNO_3_) showing the strongest catalytic effect and the lowest degradation temperatures. Among the additives, Ca_3_(PO_4_)_2_ has the weakest influence, indicating the highest relative thermal stability of the composite.

### 3.4. Biodegradation of Composites PLA

#### 3.4.1. Characteristics of Soils and Compost

Biodegradation was tested in compost and in two types of soil—field soil and horticultural soil. Table 5 presents the parameters of the soils used to test the biodegradation of composites. Field soil is acidic (pH = 5.03), garden soil is slightly acidic—close to neutral—while compost has an alkaline pH (8.68). Garden soil is the moistest soil (approximately 43.82%). Field soil, rich in heavy clay fractions, has the highest specific gravity. The highest carbon content was observed in garden soil, while nitrogen was most abundant in compost. Garden soil and compost are characterised by higher macronutrient content (calcium, potassium, sodium and magnesium) than field soil. Garden soil and compost also have higher phosphorus and sulphur content than field soil. Significantly higher manganese, iron, chromium, nickel, copper, zinc, lead and arsenic contents were observed in compost than in garden and field soil. The physicochemical properties of the tested soils and compost are likely to play a significant role in the biodegradation of PLA-based composites containing starch and mineral fertilisers. Soil pH can influence both hydrolytic and microbial degradation as alkaline conditions, like those in compost (pH 8.68), can accelerate chemical hydrolysis of ester bonds in PLA, whereas acidic soils, such as field soil (pH 5.03), may slow the process. Moisture content is also critical, since water availability facilitates polymer hydrolysis and supports microbial activity; the high moisture level in horticultural soil (~43.8%) is expected to promote faster biodegradation compared to drier soils. Additionally, the nutrient content and elemental composition of the soils may indirectly affect microbial colonisation and enzymatic activity. Higher nitrogen, phosphorus and sulphur levels in compost and horticultural soil can stimulate microbial growth, enhancing the enzymatic breakdown of PLA composites. In contrast, field soil, with low macronutrient content, may limit microbial activity and thus slow biodegradation. The presence of minerals, particularly metal ions such as Fe, Mn and Cu in compost, could also affect microbial enzyme activity, either promoting or inhibiting certain biodegradation pathways depending on concentration. Finally, soil texture and density influence aeration and water retention, which can further modulate both hydrolytic and microbial degradation rates. Overall, these factors suggest that PLA–starch–fertiliser composites degrade more rapidly in compost and horticultural soil than in field soil. Table 5 presents physicochemical parameters of soils and compost used to study the bio-degradation process of polylactide and its composites.

**Figure 12:**
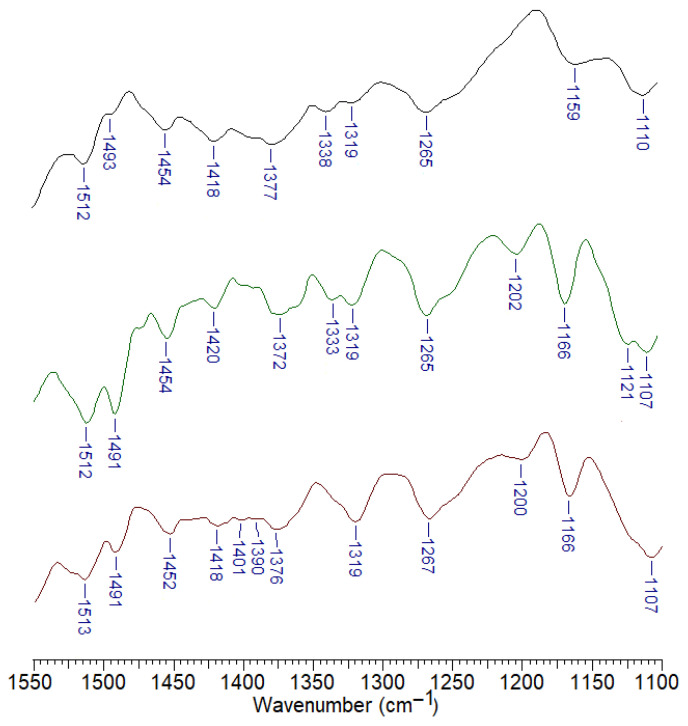
FTIR of compost (brown line), field soil (green line) and garden soil (black line) registered in the range of 1550–1100 cm^−1^.

Figure 12 shows the FTIR spectra recorded for compost, field and garden soil.

Infrared (FTIR) spectra of garden soil, field soil and compost recorded in the range of 1550–1100 cm^−1^ show clear differences in organic and mineral composition among the analysed materials. This spectral region is particularly sensitive to functional groups associated with soil organic matter as well as silicate and aluminosilicate minerals [40,41].

All samples have bands in the region of 1510–1490 cm^−1^, which are attributed to aromatic C=C stretching vibrations characteristic of humic substances and lignin-derived structures [40,42,43]. These bands are most pronounced in the compost sample, indicating a high degree of aromatic organic matter, while they appear weaker in garden soil and are least intense in field soil. Absorption bands observed at approximately 1450–1420 cm^−1^ are assigned to deformation vibrations of aliphatic C–H groups (–CH_2_ and –CH_3_) and, in part, to carbonate (${\mathrm{CO}}_{3}^{2-}$) species. In field soil, these bands are relatively sharp and intense, suggesting a higher contribution of mineral components such as carbonates. In contrast, compost displays broader bands in this region, reflecting the dominance of organic structures. Bands located around 1380–1370 cm^−1^ are associated with aliphatic C–H bending vibrations and/or symmetric stretching of carboxylate groups (${\mathrm{COO}}^{-}$), indicating the presence of organic acids and humified organic matter [44,45,46]. These signals are particularly evident in compost and garden soil, while they are less pronounced in field soil. The bands at 1335–1315 cm^−1^ and near 1265 cm^−1^ are attributed to C–O and C–N stretching vibrations typical of phenolic compounds, proteins and other nitrogen-containing organic constituents [44,47,48]. The higher intensity of these bands in compost reflects the abundance of biologically derived organic material, whereas their reduced intensity in field soil indicates a lower organic matter content. In the lower wavenumber region, between approximately 1200 and 1100 cm^−1^, strong absorption bands are observed in all samples and are assigned to Si–O–Si and Si–O–Al stretching vibrations characteristic of silicate and aluminosilicate minerals [49,50]. These bands are most intense in field soil, confirming the predominance of the mineral fraction, while their relative intensity decreases in garden soil and is lowest in compost due to dilution by organic matter.

#### 3.4.2. Biodegradation in Soils (1–5 Weeks)

**Figure 13:**
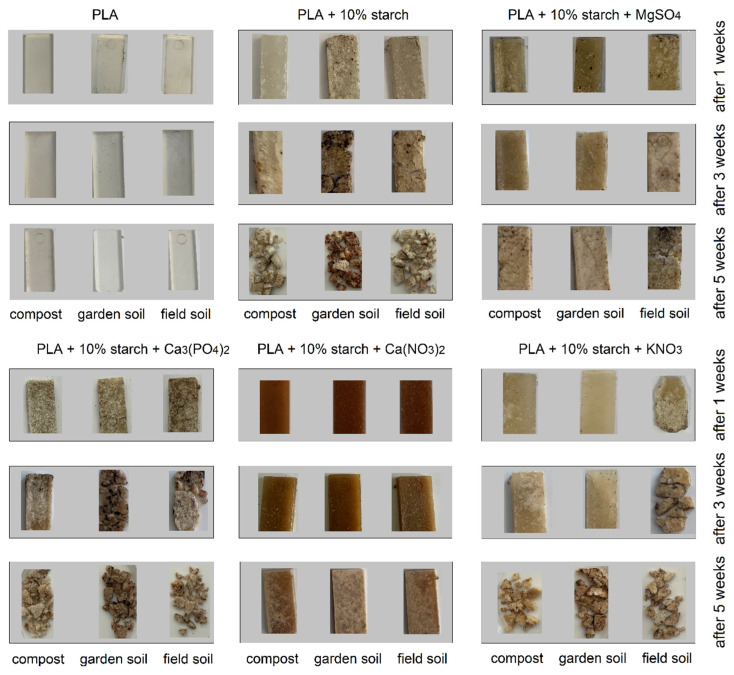
Photographs of composite samples after 1, 3 and 5 weeks of biodegradation in field soil, horticultural soil and compost.

**Figure 14:**
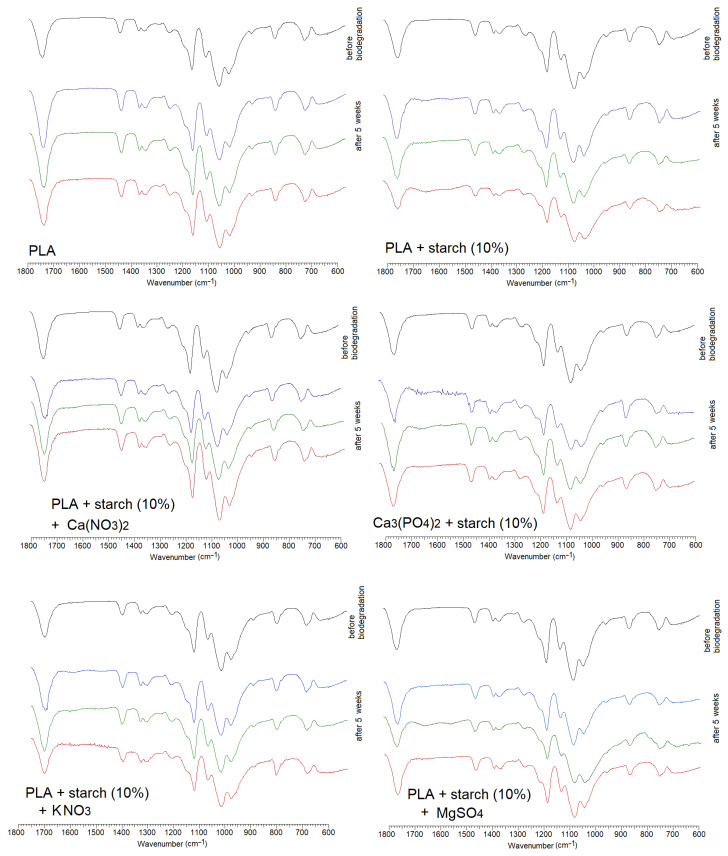
FTIR spectra for the PLA composites registered before biodegradation and after biodegradation (5 weeks) in garden soil (green line), field soil (red line) and compost (blue line).

Figure 13 shows photographs of polymers subjected to biodegradation in garden soil, field soil and compost. Observations were made after 1, 3 and 5 weeks of the process. The samples were also weighed (Table 6), and their infrared spectra (FTIR) spectra were recorded (Figure 14). Over the 5 weeks of the experiment, no significant changes in the structure of biodegradable PLA were observed in any of the soil types. The mass of the polymer did not change, and no changes were observed in the FTIR spectra compared to the polymer spectra registered before biodegradation. The PLA composite with 10% (by mass) starch additive underwent noticeable changes after just one week of the process. A slight increase in the mass of biodegraded samples in compost and field soil was observed, caused by sample swelling and water absorption from the soil. The greatest changes were observed for the sample placed in horticultural soil. After 3 weeks of the experiment, the greatest loss of mass of the biodegraded sample was observed in horticultural soil. After 5 weeks, all PLA samples mixed with starch had broken down into small fragments (no weight loss was measured). Observations show that the biodegradation process occurs fastest for the sample placed in horticultural soil. Infrared spectroscopy of the composite residues showed no significant changes in characteristic absorption bands, suggesting that although the composites underwent mass loss, the chemical structure of the remaining polymer matrix remained largely unaltered. The intensity of the stretching and deformation bands of the CH_3_ groups decreased slightly. The samples of PLA mixed with starch and MgSO_4_ did not undergo significant changes in any of the soils after 1 week. The weight of the samples increased slightly due to water absorption from the soil. After 3 weeks, polymer cracking was observed in the sample subjected to biodegradation in field soil. All samples swelled, and a slight increase in mass was observed. After 5 weeks of the process, the greatest changes were observed in the sample placed in field soil. The sample began to crack significantly, to a greater extent than the samples placed in compost and garden soil. No significant changes were observed in the FTIR spectra recorded for the PLA samples with MgSO_4_ after 5 weeks of biodegradation, except for a decrease in the intensity of the ester bond band.

The PLA samples mixed with starch and Ca_3_(PO_4_)_2_ underwent visible changes after just one week of biodegradation. In the case of compost and field soil, the samples placed there increased slightly in weight as a result of water sorption from the soil, while the sample placed in horticultural soil decreased slightly in weight. Swelling and cracking was observed in each sample. The samples became brittle. After 3 weeks of the process, the samples placed in horticultural soil and field soil cracked and fragmented. The biodegradation process in compost is slightly slower (the sample remains intact). A loss of mass was observed in all samples. After 5 weeks of biodegradation, the samples placed in each type of soil disintegrated into small fragments. The process occurs most rapidly in field soil. In the recorded infrared spectra for the samples after 5 weeks of biodegradation, slight decreases in the intensity of some bands are observed. The biodegradation rate of PLA/starch composites containing Ca_3_(PO_4_)_2_ was strongly affected by soil pH. In acidic field soil (pH = 5.3), the formation of soluble monocalcium phosphate, Ca(H_2_PO_4_)_2_, likely enhanced microbial activity, resulting in faster sample fragmentation. In neutral to slightly alkaline soils (pH 6.17–8.6), Ca_3_(PO_4_)_2_ remained largely insoluble or formed dicalcium phosphate, CaHPO_4_, slowing microbial-mediated degradation. FTIR spectra confirmed that the PLA backbone remained chemically intact, indicating that pH influenced biodegradation indirectly via phosphorus availability, rather than direct chemical interaction with PLA.

The PLA samples mixed with starch and Ca(NO_3_)_2_ did not undergo significant changes in any of the soils during the first three weeks of biodegradation. After 5 weeks, a change in the form of microcracks was observed on the surfaces of the samples. The sample biodegraded in field soil underwent the most significant changes. The spectra recorded for the samples after 5 weeks of biodegradation did not differ significantly from the spectra recorded before the biodegradation process. The samples of PLA mixed with starch and KNO_3_ underwent visible changes in the first week of the biodegradation process, especially the sample placed in field soil (which lost some of its mass). After 3 weeks of the process, a loss of mass was observed in each of the samples placed in three different types of soil. The sample biodegraded in field soil disintegrated into smaller fragments. After 5 weeks of biodegradation, each of the samples disintegrated into small fragments. The bands in the FTIR spectra recorded for the samples after disintegration (after 5 weeks) do not differ significantly from the bands present in the spectra of the samples recorded before the biodegradation process.

#### 3.4.3. Biodegradation in Soils After 9 Weeks

**Figure 15:**
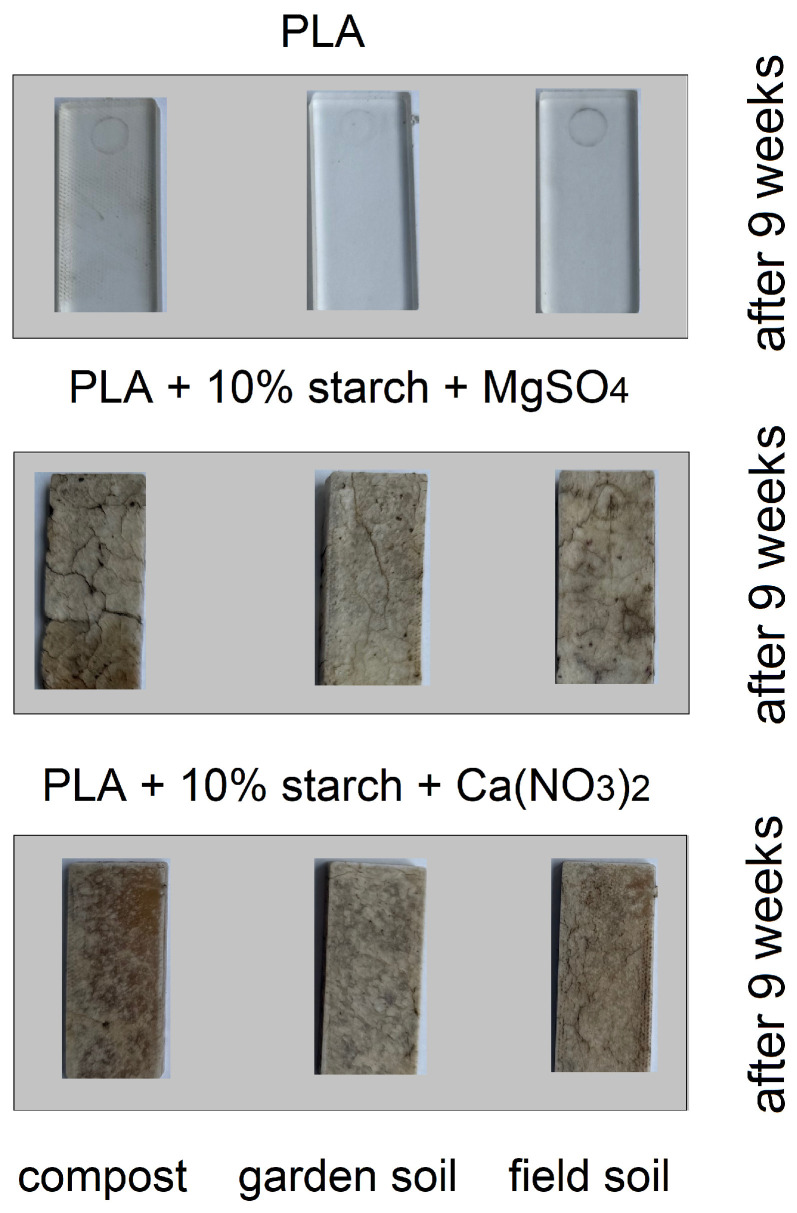
Photographs of composite samples after 9 weeks of biodegradation in field soil, garden soil and compost.

At a later stage of the biodegradation process in the three soil types, it was observed that the pure PLA samples did not change after 7 and 9 weeks. The samples retained their mass and appearance from before the biodegradation process began. No changes were observed in the FTIR spectra. The PLA samples containing starch, as well as samples containing KNO_3_ and Ca_3_(PO_4_)_2_, completely decomposed after 7 weeks of biodegradation, and the decomposition products dispersed in the soils in which they were biodegraded. The PLA samples doped with starch, Ca(NO_3_)_2_ and MgSO_4_ cracked on the surface after 7 weeks of biodegradation (Figure 15). No significant mass loss was observed in any of the soil types. The FTIR spectra recorded for these samples after 9 weeks of biodegradation showed a decrease in band intensity compared to the spectra recorded before biodegradation.

It was observed that in the case of the PLA sample doped with MgSO_4_, biodegradation occurred most rapidly in a compost environment. After 9 weeks, the sample began to disintegrate into smaller fragments. However, the PLA samples doped with Ca(NO_3_)_2_ remained intact after 9 days of biodegradation, with only surface changes observed in the samples placed in each soil type. Pure PLA did not biodegrade for at least 9 weeks in any of the soil types used in the study. The addition of starch increased the rate of polylactide degradation. Changes in composite samples are observed after just 1 week in each of the soil types used. The PLA–starch composite underwent complete biodegradation after approximately 7–9 weeks. The addition of mineral fertilisers to the PLA–starch composite can accelerate or slow down biodegradation. Among the PLA composites doped with fertilisers, PLA with KNO_3_ biodegrades the fastest. The addition of magnesium salts reduces the rate of composite degradation in each of the soil types used in the studies. The composite with calcium phosphate biodegrades faster than the PLA–starch composites. After 9 weeks, the composite was completely biodegraded. The soil that best promotes biodegradation of the tested composites is field soil. In most cases, composite degradation occurred most rapidly in this soil type.

#### 3.4.4. Biodegradability in Compost Extract

**Figure 16:**
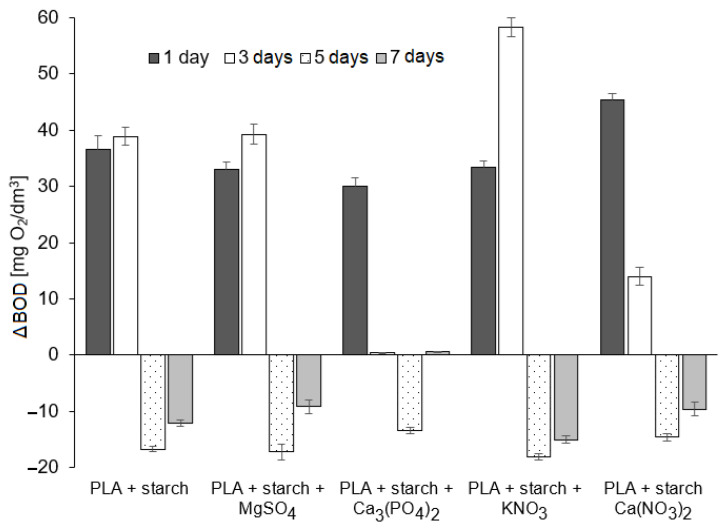
The effect of PLA composites with mineral fertilisers on the respiratory activity of the microorganisms in the compost extract (*n* = 3, $\overset{-}{x}\;\pm \;\mathrm{SD}$).

Figure 16 presents the results of the biodegradability assessment of the tested composites after 1, 3, 5 and 7 days of the experiment conducted in compost extract. The reference value for the presented results was the respiratory activity of microorganisms in pure compost extract, measured on days 1, 3, 5 and 7 of the experiment. The heights of the bars indicate changes in microbial respiratory activity (compared to the reference value) induced by the presence of the composites, measured as microbial respiratory activity in mg O_2_/dm^3^. After the first day of the experiment, it was observed that the respiratory activity of microorganisms in all samples was higher than in the pure compost solution, with the highest activity recorded for the PLA composite with Ca(NO_3_)_2_.

Considering that the respiratory activity of microorganisms in compost alone was 73 mg O_2_/dm^3^, the presence of this composite increased activity by approximately 60%. The lowest increase in activity was observed for the PLA composite with calcium phosphate—approximately 40%. After 3 days of the process, microbial activity continued to be higher in the composite samples. In the case of KNO_3_, there was an increase of approximately 30%. In the sample containing the Ca_3_PO_4_ composite, microbial activity after 3 days was comparable to that in pure compost. After 5 days of the process, microbial activity in the composite samples was lower than in the compost solution. The decreases in microbial activity in the samples containing PLA with starch, PLA with MgSO_4_ and KNO_3_ were very similar; slightly smaller decreases were observed for the PLA samples with Ca_3_PO_4_ and CaNO_3_. After 7 days, the microbial activity in the samples was still lower than that in the pure compost solution, except for the PLA with Ca_3_PO_4_ sample, where the activity was comparable to the respiratory activity of microorganisms in the compost alone.

**Figure 17:**
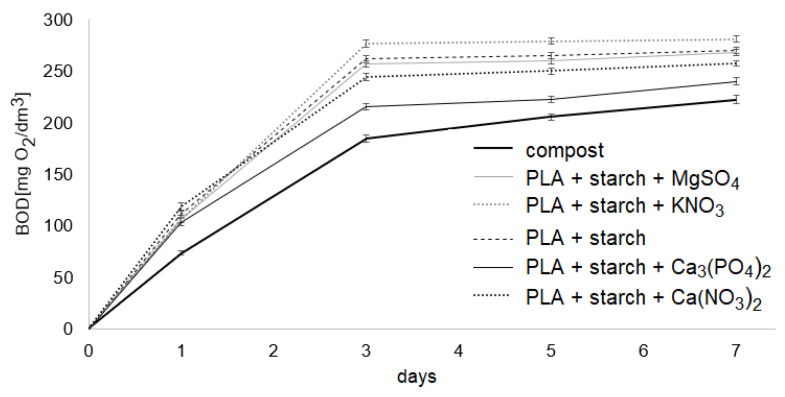
Influence of addition of polylactide composites on respiratory activity of microorganisms in the compost extract (*n* = 3, $\overset{-}{x}\;\pm \;\mathrm{SD}$).

Studies of the effect of composites on the respiratory activity of microorganisms in compost extract showed that respiratory activity in the samples with composites was higher than in the samples with the extract only, as shown in Figure 17. The changes in microbiological activity over the week of measurement differed between composite-free compost and compost extract in which composites were biodegraded. In the first three days, microbial activity in the compost containing composites was higher, while after the fifth day, the activity was lower than in the compost extract without composites. The decrease in microbial activity may result from the gradual depletion of substrates available to microorganisms or excessive concentration of metals contained in salts incorporated into the composites, released during their decomposition.

Pure PLA exhibited negligible mass loss and no significant structural changes over the test period, in agreement with previous findings that PLA degrades slowly without modification or the presence of highly active microorganisms or elevated temperatures typical of industrial composting [51,52,53]. Composites containing 10% starch displayed markedly accelerated biodegradation, with complete fragmentation observed within 7–9 weeks, which is consistent with literature reports showing that hydrophilic fillers such as starch and other natural fibres facilitate moisture penetration and microbial colonisation, thereby enhancing PLA biodegradation compared to neat PLA [54,55,56]. The addition of mineral salts modulated degradation rates: Ca(NO_3_)_2_ and MgSO_4_ slowed the process, while KNO_3_ and Ca_3_(PO_4_)_2_ accelerated it, particularly in field soil, reflecting the known role of inorganic additives as pro- or anti-degradants depending on their interactions with the polymer and microbial environment [57,58]. Respirometric measurements indicated initial stimulation of microbial activity by the composites, followed by a decline after several days, likely due to substrate depletion or ion effects, highlighting the complex interplay between filler type, environment and microbial response during PLA composite biodegradation [59,60,61].

The respirometry Biochemical Oxygen Demand (BOD) test performed in compost extract was intended as a complementary experiment to the soil burial studies, providing direct insight into the short-term microbial response to degradation products released from PLA-based composites. Unlike soil systems, which are highly heterogeneous and difficult to standardise in terms of microbial population, moisture and oxygen availability, compost extract offers a biologically active yet relatively homogeneous medium, enabling controlled assessment of microbial respiratory activity.

The choice of compost as the test medium was guided by the soil burial results, which demonstrated that compost and field soil promoted the most pronounced physical degradation and fragmentation of PLA–starch composites. This suggested that composting conditions are particularly relevant for evaluating early-stage biological utilisation of low-molecular-weight compounds released during composite disintegration. For this reason, respirometry measurements were not conducted in extracts from other soil types as the primary aim was not to compare different soils but to verify whether degradation products generated under composting conditions are biologically accessible to microorganisms.

The 7-day duration of the BOD test was selected to capture early microbial activity associated with readily biodegradable substrates, rather than long-term biodegradation kinetics. The initial increase in respiratory activity observed during the first 1–3 days indicates stimulation of microbial metabolism, likely due to the release of easily assimilable compounds originating from starch and fertiliser additives. The subsequent decrease in microbial activity relative to the compost reference suggests progressive substrate depletion and/or inhibitory effects related to the release of mineral ions from the composites. Such behaviour is characteristic of short-term respirometry assays and reflects transient microbial responses rather than sustained biodegradation processes.

The reduced microbial activity observed after several days compared to the compost reference does not contradict the soil burial results but instead complements them. While soil experiments revealed long-term physical disintegration and fragmentation of PLA composites over several weeks, the BOD test highlights that microbial utilisation of degradation products occurs primarily at early stages and may be limited by nutrient availability or ion concentration. Together, these results demonstrate that biodegradation of PLA-based composites proceeds through a combination of physical disintegration, additive-driven effects and short-term microbial activity, the relative contributions of which depend strongly on environmental conditions.

## 4. Conclusions

Polylactide is a biodegradable material, the decomposition rate of which, as shown by literature data and the research described in this article, can change with the additives used in the composites. Biodegradation of composites of PLA with starch enriched with inorganic salts clearly depended on the parameters of the medium in which they were incubated. Values of pH can be one of the factors influencing the rate of PLA biodegradation. Hydrolysis of ester bonds in the PLA polymer chain occurs faster in strongly acidic or strongly alkaline environments. After hydrolysis, polylactide is degraded by microorganisms. In a neutral environment, hydrolysis processes occur more slowly, which may initially slow down polylactide degradation. Studies have shown that the biodegradation process of the tested composites occurs most rapidly in acidic soil, which was field soil in this study. The addition of mineral salts used in the tested composites significantly influenced the biodegradation rate of the composites. Mineral compounds added to the PLA–starch composite improve its mechanical properties, besides the addition of Ca_3_(PO_4_)_2_, which deteriorate the tensile strength of the composite. Additions of mineral salts such as KNO_3_ and Ca_3_(PO_4_)_2_ significantly increase the biodegradation rate. Interestingly, the two salts differ dramatically in solubility. Ca_3_(PO_4_)_2_ is a poorly soluble salt and can form insoluble precipitates in an acidic environment. Therefore, it is not a good source of phosphorus for plants. During the biodegradation conducted in this experiment, the acidic environment of field soil caused the precipitation of poorly soluble precipitates, which could be observed on the biodegradable composites. The presence of these precipitates and their interaction with water likely contributed to increased swelling and surface damage of the composites, which accelerated the mechanical degradation of the material. Potassium nitrate salt is highly soluble and can also be a source of potassium and nitrates for plants, but it also serves as a nutrient for the microorganisms involved in PLA degradation. Respiratory activity studies showed that microorganisms exhibited increased respiratory activity in the presence of PLA with KNO_3_. Thus, the enhanced biodegradation of PLA/KNO_3_ composites can be attributed not only to physicochemical effects but also to the stimulation of microbial activity. Magnesium sulphate and calcium nitrate are highly water-soluble and neutral salts; however, the addition of these salts reduced the biodegradation rate of PLA. Decomposition of these composites began approximately 10 weeks after exposure to soil. This delayed degradation suggests that these salts do not promote microbial activity or structural weakening of the composite to the same extent as KNO_3_ or Ca_3_(PO_4_)_2_. The faster biodegradation of composites in field (acidic) soil can be explained by the hydrolysis of starch contained in the composites (10%). An acidic environment favours the starch hydrolysis process, which influences composite degradation and further biodegradation. Another important factor in polylactide biodegradation is the degree of polymer fragmentation; in this case, the thickness of the biodegraded samples, which was 4 mm, was crucial. A faster biodegradation rate could be expected with thinner samples. Therefore, the observed biodegradation rates should be interpreted with consideration of sample geometry and diffusion limitations. It should also be noted that the addition of mineral salts to the prepared composites did not affect the chemical structure of polylactide. The FTIR and Raman spectra of the prepared composites did not differ from those of pure PLA. These analyses were performed to verify the chemical stability of PLA during high-temperature processing (>180 °C) in the presence of inorganic salts and to exclude the formation of new chemical bonds or degradation products. The addition of mineral salts to PLA also did not significantly affect its thermal properties, as demonstrated by DSC and TG thermal analysis. This confirms that the observed differences in biodegradation behaviour result from physical effects and environmental interactions rather than from changes in the chemical structure of the polymer.

## Figures and Tables

**Table 1:** Tensile strength measurements of PLA composites.

	$\mathrm{Tensile}\;\mathrm{Strength}\;\sigma_{M}$ [MPa]	σ	Elongation at Tensile Strength $\varepsilon_{M}$ [%]	σ	Stress at Failure $\sigma_{B}$ [MPa]	σ	Elongation at Failure $\varepsilon_{B}$ [%]	σ
PLA	67.77	0.53	1.53	0.05	23.83	1.39	1.77	0.17
PLA + starch (10%)	35.33	1.43	0.73	0.05	13.80	0.54	0.73	0.05
PLA + starch (10%) + Ca(NO_3_)_2_	48.23	1.16	0.93	0.05	18.57	0.04	0.97	0.05
PLA + starch (10%) + MgSO_4_	36.10	1.02	0.77	0.09	14.10	1.84	0.77	0.09
PLA + starch (10%) + KNO_3_	40.10	0.37	0.80	0.00	15.67	0.21	0.80	0.00
PLA + starch (10%) + Ca_3_(PO_4_)_2_	29.13	1.60	0.63	0.05	11.37	1.01	0.67	0.05

σ—standard deviation.

**Table 2:** Wavenumbers of bands present in FTIR, ATR, and Raman spectra for PLA composites, along with their assignment.

PLA		PLA + 10% Starch		PLA + 10% Starch + MgSO_4_		PLA + 10% Starch + KNO_3_		PLA+ 10% Starch + Ca_3_(PO_4_)_2_		PLA+ 10% Starch + Ca(NO_3_)_2_		Assignments
FTIR	Raman	FTIR	Raman	FTIR	Raman	FTIR	Raman	FTIR	Raman	FTIR	Raman	
3001 w	3002 vs	2995 w	3001 s	3000 w	3003 s	3001 w	3001 s	2999 w	3001 s	2998 w	3001 s	ν_as_CH_3_
-	2947 vs	-	2946 vs	-	2947 vs	-	2947 vs	-	2946 vs	-	2946 vs	ν_as_CH_3_
2930 w	-	2921 w	-	2925 w	2907 m	2927 w	-	2923 w	-	2925 w	-	ν_as_CH_3_
1750 s	1771 m	1746 s	1770 s	1749 s	1774 s	1744 s	1771 s	1745 s	1767 s	1748 s	1770 s	νC=O
1456 m	1455 m	1451 m	1453 m	1455 m	1456 m	1450 m	1455 s	1450 m	1456 s	1452 m	1455 m	νCH_3_
1383 m	1390 w	1382 m	1385 m	1383 m	1386 m	1381 m	1391 m	1381 m	1390 m	1374 m	1385 m	defCH_3_
1361 m	1360 w	1359 m	1361 m	1361 m	1354 m	1359 m	-	1358 m	1345 m	1360 m	1361 m	νCH-CH_3_
-	1298 m	-	1298 m	-	1298 m	-	1299 m	-	1300 m	-	1300 m	νCH + νC-O-C
1266 m	1257 w	1259 m	-	1266 m	1262 w	1262 m	1270 w	1260 m	1260 w	1266 m	-	νCH + νC-O-C
1181 s	1184 w	1180 s	1183 m	1181 s	1182 w	1180 s	-	1180 s	1190 w	1180 s	-	defC-O-C
1128 m	1129 m	1128 s	1129 m	1128 s	1131 m	1128 m	1128 m	1127 s	1131 m	1128 s	1136 m	defCH_3_
1078 vs	1098 m	1077 vs	-	1079 vs	-	1076 vs	-	1077 vs	-	1078 vs	-	defC-O-C
1041 s	1044 m	1039 s	1045 m	1041 s	1046 m	1039 s	1046 m	1039 s	-	1040 s	1043 m	νCH-CH_3_
867 m	874 w	866 m	874 s	867 m	874 s	865 m	873 s	866 m	873 s	867 m	875 s	νC-CCO
754 m	761 w	754 m	737 w	754 m	753 w	754 m	743 w	753 m	739 m	754 m	-	νC=O
698 m	697 w	698 m	-	700 m	691 w	698 m	704 w	698 m	695 w	699 m	-	νC=O

def—deformation; ν—stretching; as—asymmetric; intensities: vs—very strong; s—strong; m—medium; w—weak.

**Table 3:** Maxima on DSC curves for polylactide and its composites.

	DSC_peak1_ [°C]	Process	DSC_peak2_ [°C]	Process	DSC_peak3_ [°C]	Process
PLA	65.0	vitrification	82.5	crystallisation	170.0	melting
PLA + starch	62.5	vitrification	82.5	crystallisation	170.0	melting
PLA + starch + KNO_3_	62.5	vitrification	92.5	crystallisation	170.0	melting
PLA + starch + MgSO_4_	62.5	vitrification	85.0	crystallisation	170.0	melting
PLA + starch + Ca_3_(PO_4_)_2_	62.5	vitrification	97.5	crystallisation	167.5	melting
PLA + starch + Ca(NO_3_)_2_	62.5	vitrification	95.0	crystallisation	170.0	melting

**Table 4:** Thermoanalytical results (TG, DTG) for polylactide and their composites.

	Stage	Temp. Range [°C]	DTG_max_ [°C]	Loss Mass [%]
PLA	decomposition	290–380	362	96.50
	burning	390–500	-	99.20
PLA + starch	decomposition	275–390	353	97.15
	burning	400–500	-	98.45
PLA + starch + KNO_3_	decomposition/ dehydration	50–160	154	2.70
	decomposition	260–327	301	89.95
	burning	330–500	-	94.85
PLA + starch + MgSO_4_	decomposition/ dehydration	60–180	171	5.65
	decomposition	260–370	353	93.25
	burning	380–500	-	97.50
PLA + starch + Ca_3_(PO_4_)_2_	decomposition/ dehydration	50–180	162	8.45
	decomposition	250–370	320	95.50
	burning	-	-	97.15
PLA + starch + Ca(NO_3_)_2_	decomposition/ dehydration	60–215	154	4.35
	decomposition	260–360	293	93.15
	burning	370–500	-	95.85

**Table 5:** Physicochemical properties elemental analysis of compost and field soil and garden soil.

	Compost	Field Soil	Garden Soil
Physicochemical Properties			
pH	8.68 ± 0.07	5.03 ± 0.06	6.17 ± 0.02
pH (KCl)	7.27 ± 0.05	4.38 ± 0.03	5.83 ± 0.02
Humidity [%]	24.82 ± 0.79	22.50 ± 0.21	43.82 ± 0.22
Specific gravity * [g/cm^3^]	1.426 ± 0.083	2.620 ± 0.110	1.066 ± 0.011
Specific gravity ** [g/cm^3^]	1.442 ± 0.056	2.590 ± 0.080	0.900 ± 0.027
Elemental Analysis			
Carbon [% d.m]	18.69 ± 0.46	1.06 ± 0.03	11.84 ± 0.24
Nitrogen [% d.m]	2.25 ± 0.09	0.11 ± 0.01	0.48 ± 0.03
Ca [mg/g]	14.87 ± 1.21	<LOD	25.64 ± 1.22
K [mg/g]	6.86 ± 1.03	0.61 ± 0.03	2.00 ± 0.02
Na [mg/g]	0.71 ± 0.04	0.10 ± 0.02	0.28 ± 0.04
Mg [mg/g]	2.88 ± 0.53	0.08 ± 0.01	1.95 ± 0.24
P [μg/g]	1952.89 ± 1.62	357.48 ± 1.31	1170.04 ± 2.93
S [μg/g]	1158.59 ± 3.08	48.01 ± 1.71	1887.06 ± 0.95
Cl [μg/g]	363.90 ± 1.21	333.50 ± 6.83	477.35 ± 2.52
Cr [μg/g]	48.17 ± 2.34	16.92 ± 2.73	4.92 ± 0.19
Mn [μg/g]	249.41 ± 5.42	102.81 ± 2.87	50.71 ± 0.82
Fe [μg/g]	6598.88 ± 1.15	2373.28 ± 0.85	2206.60 ± 1.38
Ni [μg/g]	14.13 ± 2.45	7.03 ± 0.78	1.86 ± 0.3
Cu [μg/g]	24.66 ± 1.31	2.91 ± 0.87	14.65 ± 1.31
Zn [μg/g]	156.11 ± 1.21	28.39 ± 2.05	29.76 ± 1.12
As [μg/g]	12.01 ± 1.16	4.15 ± 1.07	3.34 ± 0.74
Pb [μg/g]	32.12 ± 1.69	11.52 ± 1.06	8.80 ± 0.78

* pycnometric method. ** determined using the La Chatelier flask.

**Table 6:** Loss of composite mass during biodegradation.

Composite	Soil Type		After 1 Week		After 3 Weeks		After 5 Weeks
		Δm [g]	σ	Δm [g]	σ	Δm [g]	σ
PLA	Compost	-	-	-	-	-	-
	Garden Soil	-	-	-	-	-	-
	Field Soil	-	-	-	-	-	-
PLA + 10% starch	Compost	0.0434	0.0087	−0.1724	0.1444	Disintegration	Disintegration
	Garden Soil	−0.0370	0.0163	−1.4122	0.0801	Disintegration	Disintegration
	Field Soil	0.0539	0.0133	0.1473	0.1211	Disintegration	Disintegration
PLA + 10% starch + MgSO_4_	Compost	0.0551	0.0146	0.1476	0.0633	0.2299	0.0751
	Garden Soil	0.0793	0.0268	0.2210	0.0266	0.2541	0.0233
	Field Soil	0.0793	0.0058	0.1163	0.0322	0.0861	0.0407
PLA + 10% starch + KNO_3_	Compost	0.0374	0.0151	−0.4913	0.0246	Disintegration	Disintegration
	Garden Soil	0.2286	0.1461	−0.2193	0.1621	Disintegration	Disintegration
	Field Soil	−0.2330	0.1182	−0.4913	0.2046	Disintegration	Disintegration
PLA + 10% starch + Ca_3_(PO_4_)_2_	Compost	0.1283	0.0111	−0.2195	0.0742	Disintegration	Disintegration
	Garden Soil	−0.7715	0.2519	−1.0560	0.2080	Disintegration	Disintegration
	Field Soil	0.1790	0.0228	−1.1648	0.1645	−1.3438	0.1836
PLA + 10% starch + Ca(NO_3_)_2_	Compost	0.0569	0.0046	0.0896	0.0237	0.1589	0.0195
	Garden Soil	0.0697	0.0021	0.1162	0.0083	0.1723	0.0347
	Field Soil	0.0569	0.0046	0.0895	0.0237	0.1590	0.0195

## Data Availability

The data presented in this study are available upon request from the corresponding author.
